# Fostering public climate change discussions from a social interaction perspective

**DOI:** 10.3389/fpsyg.2023.1258150

**Published:** 2023-09-05

**Authors:** Jianchi Tian, Xiaoqing Zheng, Yan Sun

**Affiliations:** ^1^Key Laboratory of Behavioral Science, Institute of Psychology, Chinese Academy of Sciences, Beijing, China; ^2^Department of Psychology, University of Chinese Academy of Sciences, Beijing, China

**Keywords:** climate change discussion, social interaction, social norm, response efficacy, self-efficacy

## Abstract

Public discussions on climate change, as a form of social interaction, are widely recognized as effective tools for promoting collective action. However, there is limited research on examining the factors that influence climate change discussions from a social interaction perspective. In the present study, we conducted a large sample (*N* = 1,169) survey to investigate personal (such as self-efficacy and personal response efficacy) and others' (such as perceived others' response efficacy and social norms) factors influencing climate change discussions from a social interaction perspective. The results showed that (i) for people with high climate change perceptions, personal response efficacy, self-efficacy, and social norms have positive effects on climate change discussions, but the effect of perceived others' response efficacy on climate change discussion is not significant; (ii) for people with low climate change perceptions, self-efficacy and social norms have positive effects on climate change discussions, but the effects of personal response efficacy and perceived others' response efficacy on climate change discussion are not significant; (iii) irrespective of individuals' high or low perceptions of climate change, social norm remains the most important predictor of climate change discussions. These findings make valuable contributions to the theoretical literature and intervention efforts regarding climate change discussions from a social interaction perspective.

## 1. Introduction

Climate change poses a significant challenge to human society, impacting both human health and socio-economic conditions (Berkhout et al., 2002; Patz et al., 2005). Unfortunately, insufficient collective action has allowed carbon emissions to persist at high levels (United Nations Environment Programme., 2020), exacerbating the problem. Thus, it is crucial that we urgently come together and take collective action to mitigate climate change.

Researchers have recognized that public discourse on social issues, particularly interpersonal communication, plays a vital role in fostering civic engagement regarding climate change (Swim et al., 2018). Empirical studies demonstrate that engaging in public discussions about climate change helps reinforce the importance of mitigation and adaptation (Clayton et al., 2015; Goldberg et al., 2019), thereby encouraging people to participate in collective action. However, surveys indicate that only a minority of individuals actively engage in interpersonal climate change discussions (Leiserowitz et al., 2015, 2019). Even formal education settings like schools and informal settings such as aquariums often fail to adequately address this issue (Swim and Fraser, 2013; Plutzer et al., 2016). This lack of engagement is concerning because if the majority remains indifferent to climate change or continues unsustainable behaviors, mitigating climate change solely through the actions of a few individuals becomes exceedingly challenging. Therefore, it is imperative to promote public concern and participation in climate change.

Recent studies have introduced various interventions, such as education, to encourage public participation in climate change discussions (Geiger et al., 2017). These interventions are based on the premise that people refrain from discussing climate change due to negative self-assessments. One barrier is the limited scientific understanding of climate change (Swim et al., 2017), which encompasses beliefs in its occurrence, human causation, worry about its impacts, and perceptions of associated risks (Tian et al., 2022). Enhancing scientific knowledge has been shown to effectively promote participation in climate change discussions (Swim et al., 2017). Additionally, individuals' belief in the efficacy of their own contributions to discussions and their self-perceived ability to engage in such conversations can impede participation (Bandura, 1977, 1982). If someone believes that discussing climate change will not yield the desired positive outcomes, or they feel incapable of participating in such discussions, they are likely to abstain from engaging in conversations about climate change (Swim et al., 2014). Research indicates that interventions that increase knowledge about climate change also enhance individuals' self-efficacy and response efficacy, leading to more frequent discussions (Geiger et al., 2017).

However, climate change discussions differ from traditional top-down communication in that they involve social interaction (Goldberg et al., 2019). These discussions are not one-way but rather two-way or even multi-way exchanges. People construct their understanding of climate change through social interaction and develop a shared reality or perspective (Swim et al., 2018). Consequently, the lack of climate change discussions may stem from socially constructed silence (Geiger and Swim, 2016), along with individuals' negative self-assessments. Socially constructed silence arises when people anticipate others' views and actions before initiating discussions. For instance, the spiral of silence theory suggests that individuals are less inclined to express their opinions if they believe their viewpoints are in the minority and that the majority holds opposing views (Noelle-Neumann, 1991, 1993). Moreover, individuals may avoid engaging in climate change discussions to manage impressions, fearing disapproval (Sechrist et al., 2004) or loss of respect (Stangor et al., 2003).

While self-assessment and socially constructed silence provide insights into why people refrain from climate change discussions based on their assessment of others, there are certain limitations. Since climate change discussions involve two interacting parties—the self and the other—we can deconstruct these discussions into two perspectives: self-assessment and assessment of others. Existing research primarily focuses on individuals assessing the personal benefits of participating in discussions (e.g., Geiger et al., 2017). However, since discussions are inherently social interactions (Swim et al., 2018), if an individual's participation fails to contribute to the other person's knowledge, they may feel helpless (Maier and Seligman, 1976). Therefore, examining response efficacy solely from a self-assessment perspective overlooks the interactive nature of discussions. Additionally, we propose that social norms play a significant but often overlooked role in social interactions (Morris et al., 2015). Social norms encompass shared beliefs and behaviors within a group, and people seek common ground during discussions (Kashima, 2014). The success of interaction and dialogue hinges on prevalent behaviors within the environment (Leung and Morris, 2015). In other words, social norms largely determine the sustainability of certain behaviors in social interactions. If a behavioral norm surrounding climate change discussions does not exist within a particular group, it becomes challenging for individuals to engage in such discussions (Morris et al., 2015). In summary, previous research has not adequately addressed why people refrain from climate change discussions from a social interaction perspective. Consequently, this study utilizes a large survey sample to explore the impact of response efficacy and social norms on climate change discussions from a social interaction perspective, comparing them to the effects of self-efficacy and response efficacy from a self-assessment viewpoint.

### 1.1. Perception of climate change

The understanding of climate change plays a crucial role in motivating people to engage in collective action, encompassing beliefs and emotions (Tian et al., 2022). The belief aspect comprises notions about climate change, human-caused climate change, and assessments of climate change risk (Tian et al., 2022). Meteorological evidence indicates that the global climate is changing (Hoegh-Guldberg et al., 2019), and climate scientists widely agree that human activities contribute to global warming (IPCC., 2021; Lynas et al., 2021). However, national surveys data revealed that scientific knowledge regarding climate change has been lacking in recent years. In 2013, only 42% of U.S. adults believed that “most scientists think global warming is happening.” As of November 2019, only 55% of U.S. adults were believed to hold this belief (Leiserowitz et al., 2020). False perceptions and misconceptions hinder the acceptance of human-caused climate change and its severe consequences (Van Stekelenburg et al., 2021). These misperceptions prevent individuals from connecting extreme weather events to climate change and impede their engagement in climate change discussions (Boudet et al., 2020). Additionally, the emotional dimension, which usually refers to people's concerns about climate change, is an individual's emotional state characterized by repeated experiences of climate anxiety thoughts (Bouman et al., 2020). Climate change worried individuals tend to engage in the topic of climate change and feel disturbed by the consequences of climate change (Van der Linden, 2017; Bouman et al., 2020). This emotional state often connects individuals to the abstraction of climate change and guides people to engage in mitigation actions (Van der Linden, 2017). Research has shown that participation in climate change education programs increases individuals' worries about climate change and their belief in their ability to engage in discussions on the topic (Swim et al., 2017). Thus, the following hypotheses are proposed:

**H1:** There is a significant difference between low and high climate change perceptions groups on climate change discussions. Specifically, compared to low climate change perceptions group, high climate change perceptions group are more likely to engage in climate change discussions.

### 1.2. Self-efficacy

Self-efficacy, an essential component of Social Cognitive Theory, refers to individuals' beliefs in their abilities to achieve specific behavioral goals in a particular domain (Bandura, 1977, 1986). Self-efficacy influences people's choices of activities and social environments and shapes their behavior (Bandura, 1997). High self-efficacy fosters positive attitudes and actions as individuals perceive themselves as capable of coping with challenges. Conversely, low self-efficacy leads people to avoid tasks or situations beyond their perceived capabilities and focus on reducing emotional distress. When applied to climate change discussions, individuals are more inclined to engage in these discussions when they believe in their capacity to do so. Previous research supports the idea that self-efficacy facilitates climate change discussions. A natural experimental study revealed that exposure to climate change informational interventions increased individuals' self-efficacy and, consequently, led to more frequent discussions about climate change (Geiger et al., 2017). Thus, we propose the following hypotheses:

**H2:** Self-efficacy has a positive influence on climate change discussions.

### 1.3. Response efficacy

According to Social Cognitive Theory (Bandura, 1986), outcome expectations play a significant role in motivating individuals to engage in certain behaviors. Individuals who believe that their actions will yield positive outcomes (i.e., high response efficacy) are more likely to invest effort in initiating and sustaining those behaviors compared to individuals with low response efficacy. In public climate action, individual response efficacy was considered the perceived impact of one's cooperative behavior on the collective outcome (Doherty and Webler, 2016). When considering social interactions, the assessment of behavioral outcomes should encompass both personal and others' effects. In the context of climate change discussions, we propose that response efficacy refers to an individual's belief that engaging in these discussions can result in meaningful progress or action. It involves the perception that participating in conversations about climate change can lead to positive outcomes that benefit not only oneself but also others. However, existing research has primarily focused on personal response efficacy (e.g., Geiger et al., 2017), overlooking the importance of considering others in social interactions. Differentiating objects in social interactions is crucial since discussing climate change may not be perceived as useful if it does not benefit the other party in discussion. In this study, we examine two types of response efficacy—personal and others'—and compare their relevance to climate change discussions within the framework of social interaction. To sum up, the following hypotheses are proposed:

**H3:** Response efficacy (including perceived personal and others' response efficacy) has a positive effect on climate change discussions.

### 1.4. Social norms

Social norms are influential predictors of behavior, governing group dynamics (Morris et al., 2015). To avoid social exclusion and maintain group cohesion, individuals tend to conform to the majority's behavior (Schneider and van der Linden, 2023). Numerous studies have demonstrated that social norms, particularly descriptive norms, influence environmental behaviors such as public transportation use (Heath and Gifford, 2002), energy conservation (Nolan et al., 2008; Bonan et al., 2020), and recycling (Schultz, 1999; Liu et al., 2022). These findings support the hypothesis that people are more likely to participate in a behavior when they perceive others engaging in it (Doherty and Webler, 2016). According to Social Cognitive Theory (Bandura, 1986), social norms provide evidence of others' efficacy beliefs when individuals face uncertain situations. While social norms are commonly categorized as descriptive, imperative, and dynamic (Schneider and van der Linden, 2023), empirical evidence suggests that descriptive norms have a stronger and more enduring impact (Doherty and Webler, 2016). In the context of climate change discussions, the behavior of others reflects their ability to engage in such discussions. Social norms reflect behavioral standards and the reality of others' actions, enabling individuals to assess others' self-efficacy based on observed behaviors. However, due to limited research on climate change discussions, there is a lack of studies considering the role of social norms from a social interaction perspective. Given the significance of social norms in influencing social interactions, we propose that:

**H4:** social norms can enhance individuals' willingness to engage in climate change discussions.

## 2. Methods

### 2.1. Data collection

The participants were recruited on the Credamo platform, which serves as an online data collection platform. Credamo boasts an online sample repository of over three million in China. Consequently, we employed a convenience sampling method to recruit 1,169 valid respondents (M_age_ = 33.03, SD = 7.54; 46.9% females, *N* = 548; 53.1% males, *N* = 621) nationwide. We assessed the frequency of climate change discussions, self-efficacy, response efficacy, social norms, and climate change perceptions of the respondents and collected their sociodemographic information (shown in Table 1).

**Table 1:** Socio-demographic information of respondents.

**Characteristic Socio-demographic**		**Frequency (%)**
Gender	Male	621 (53.1%)
	Female	548 (46.9%)
Place of residence	Rural	130 (11.1%)
	Urban	1,039 (88.9%)
Income (RMB Yuan/Month)	Under 2,000	160 (13.7%)
	2,000-4,999	260 (22.2%)
	5,000-9,999	517 (44.2%)
	10,000-19,999	195 (16.7%)
	Above 20,000	37 (3.2%)
Education	Middle school and below	21 (1.8%)
	High school/secondary	106 (9.1%)
	Bachelor's degree/junior college	952 (81.4%)
	Graduate students	90 (7.7%)

### 2.2. Measurement of variables

#### 2.2.1. Climate change discussion

Following the International Public Opinion on Climate Change report (Leiserowitz et al., 2022), we assessed climate change discussions by posing the question, “How often do you typically discuss environment-related topics with your family or friends?” Participants rated their response on an 11-point scale ranging from 0 (never discuss) to 10 (always discuss).

#### 2.2.2. Self-efficacy

We gauged self-efficacy by utilizing a statement adapted from Geiger et al.'s (2017) study: “I possess sufficient knowledge about environmentally related topics to engage in discussions with my family or friends.” Participants rated their agreement on an 11-point scale, where 0 indicated strong disagreement and 10 denoted strong agreement.

#### 2.2.3. Response efficacy

In this study, we differentiated response efficacy into two aspects: personal response efficacy, which refers to an individual's perception of the discussion's usefulness for oneself, and others' response efficacy, which pertains to its usefulness for others. For self-response efficacy, we employed two statements modified from Geiger et al.'s (2017) research: “Engaging in the discussion can alter my own perspectives on environmental issues” and “Engaging in the discussion can encourage my personal environmental behavior.” Similarly, for others' response efficacy, we utilized two adapted statements: “Engaging in discussions can change my family's or friends' perceptions of environmental issues” and “Engaging in discussions can promote my family's or friends' environmental behaviors.” All four questions assessing response efficacy were evaluated on an 11-point scale, ranging from 0 (strongly disagree) to 10 (strongly agree).

#### 2.2.4. Social norms

Social norms (specifically, descriptive norms) are usually designed by researchers as a percentage of participation in a behavior such as 75% (Cialdini et al., 1990; Cialdini and Jacobson, 2021). To measure social norms, respondents were asked to indicate, on an 11-point scale, the extent to which their family members or friends engage in environment-related discussions. A score of 0 indicated very little involvement, while a score of 10 represented significant involvement.

#### 2.2.5. Climate change perception

Climate change perceptions include climate change beliefs, beliefs in human causation, climate change risk perceptions, and climate change worry. We assessed respondents' climate change perceptions using items from the International Public Opinion on Climate Change report (Leiserowitz et al., 2022). Climate change beliefs were measured by a statement - “I think climate change is happening” - on an 11-point scale (0 = strongly disagree, 10 = strongly agree). Beliefs in human causation were measured through one statement (0 = climate change is caused by natural causes, 10 = climate change is caused by human-caused). Perceived risk of climate change was measured through three questions (Cronbach's α = 0.85), one of which was: 'How much of a global impact do you think climate change will have?' (0 = very little, 10 = very much)“, and for the other two questions we replaced global with China and the region where you live. One question was used to measure climate change worry, namely ”How worried are you about climate change? (0 = not worried at all, 10 = very worried)“.

#### 2.2.6. Control variables

We collected socio-demographic information on all respondents, including gender, age, place of residence, monthly income and education level. Gender (male = 1, female = 0), place of residence (urban = 1, rural = 0), monthly income (Income 1: <2,000 Yuan = 1, not <2,000 Yuan = 0; Income 2: 2,000-4,999 Yuan = 1, not 2,000-4,999 Yuan = 0; Income 3: 5,000-9,999 Yuan = 1, not 5,000-9,999 Yuan = 0; Income 4: 10,000-19,999 Yuan = 1, not 10,000-19,999 Yuan = 0; Income 5: above 20,000 Yuan = 1, not above 20,000 Yuan = 1) and education (education 1: middle school and below = 1, non-middle school and below = 0; education 2: high school/secondary = 1, non-high school/secondary = 0; education 3: bachelor's degree/junior college = 1, non-bachelor's degree/junior college = 0; education 4: graduate students = 1, non-graduate students = 0) were coded as dummy variables, and age was coded as a continuous variable (for a similar method, see Tian et al., 2022).

## 3. Results

### 3.1. Groups with high and low climate change perceptions

To investigate the impact of climate change perceptions on discussions, we conducted K-means cluster analysis to categorize respondents into two groups: high (*N* = 676) and low (*N* = 493) climate change perception. This categorization helps us understand the psychological mechanisms underlying climate change discussions among individuals with different levels of knowledge and enables us to tailor interventions accordingly. Table 2 presents the results of a *t*-test, which demonstrate that the group with high climate change perception exhibited significantly stronger beliefs in climate change, beliefs in human causation, risk perceptions of climate change, and climate change worry compared to the group with low climate change perception. Importantly, there was also a significant difference in the frequency of climate change discussions between the two groups, with individuals possessing high climate change perceptions engaging in discussions more frequently than those with low climate change perceptions. Considering that climate change perception involves individuals' comprehension and evaluation of climate change (Tian et al., 2022), our findings additionally indicate variations among groups with differing levels of climate change perception in terms of their beliefs, risk perceptions, and apprehensions regarding climate change. As a result, high climate change perceptions generally signify that individuals possess stronger convictions about the existence of climate change, attributions of human influence, evaluations of associated risks, and levels of worry, when contrasted with those exhibiting lower levels of climate change perception.

**Table 2:** The differences between groups with high and low climate change perceptions.

	**Group**	**M ±SD**	***t* (df)**	***P***
Belief in climate change	High	9.18 ± 0.89	24.65 (841.85)	< 0.001
	Low	7.57 ± 1.24		
Belief in human causation	High	8.65 ± 1.06	22.64 (847.08)	< 0.001
	Low	6.89 ± 1.46		
Climate change worry	High	8.83 ± 1.01	30.34 (862.15)	< 0.001
	Low	6.71 ± 1.37		
Risk perceptions of climate change	High	8.83 ± 0.84	29.49 (900.29)	< 0.001
	Low	7.11 ± 1.07		
Discussions	High	7.32 ± 1.92	11.79 (1167)	< 0.001
	Low	5.94 ± 2.07		

### 3.2. Factors influencing climate change discussions in groups with high climate change perceptions

We employed multiple linear regression models, utilizing a stepwise approach, to identify factors influencing climate change discussions within the group characterized by high climate change perceptions. Through multilevel linear regression analysis, we developed three models. In Model 1, we examined the influence of sociodemographic variables on climate change discussions. As shown in Table 3, the results revealed that Income 1 had a significant negative association with climate change discussion, while Education 1, Education 2, and Education 3 were positively associated with climate change discussion. Specifically, participants with a monthly income below 2,000 Yuan were more likely to engage in climate change discussions (β = −0.27, se = 0.47, *p* < 0.001). Moreover, individuals with a junior high school education or below (β = 0.12, se = 0.60, *p* = 0.007), individuals with a high school/vocational school education (β = 0.14, se = 0.37, *p* = 0.02), and individuals with a bachelor's degree/college education (β = 0.18, se = 0.28, *p* = 0.001) are more likely to engage in discussions about climate change.

**Table 3:** Factors influencing climate change discussions in groups with high climate change perceptions.

	**Model 1**			**Model 2**			**Model 3**		
	**β**	**SE**	***p***	**β**	**SE**	***p***	**β**	**SE**	***p***
Gender	−0.02	0.15	0.56	−0.02	0.12	0.44	−0.01	0.08	0.54
Age	0.03	0.01	0.46	0.01	0.01	0.77	0.00	0.01	0.94
Place of residence	0.00	0.25	0.96	0.00	0.20	0.99	0.00	0.14	0.95
Income 1	−0.27	0.47	0.00	−0.14	0.38	0.01	0.04	0.27	0.30
Income 2	−0.12	0.41	0.17	−0.10	0.33	0.14	0.06	0.24	0.26
Income 3	−0.14	0.39	0.17	−0.13	0.32	0.10	0.04	0.22	0.54
Income 4	−0.10	0.41	0.23	−0.07	0.33	0.31	0.05	0.23	0.34
Education 1	0.12	0.60	0.01	0.05	0.48	0.16	0.03	0.34	0.25
Education 2	0.14	0.37	0.02	0.06	0.30	0.16	0.04	0.21	0.19
Education 3	0.18	0.28	0.00	0.09	0.22	0.05	0.07	0.16	0.02
Personal response efficacy				0.29	0.07	0.00	0.13	0.05	0.00
Perceived others' response efficacy				0.34	0.07	0.00	0.00	0.05	0.92
Personal efficacy							0.23	0.04	0.00
Social norm							0.58	0.03	0.00
R^2^	0.09			0.44			0.69		
F	5.95			32.82			79.28		

β, standardized; SE, standard error.

In Model 2, after controlling for sociodemographic variables, we investigated the effect of response efficacy on climate change discussions. The results indicated that both perceived personal (β = 0.29, se = 0.07, *p* < 0.001) and others' (β = 0.34, se = 0.07, *p* < 0.001) response efficacy positively predicted climate change discussions. In Model 3, building upon Model 2, we explored the predictive effects of self-efficacy and social norms on climate change discussions. The findings revealed that both self-efficacy (β = 0.23, se = 0.04, *p* < 0.001) and social norms (β = 0.58, se = 0.03, *p* < 0.001) had a positive influence on climate change discussions. However, the predictive effect of perceived others' response efficacy in Model 3 was not significant (*p* > 0.05).

In summary, Model 3, which incorporated self-efficacy and social norms, accounted for 69% of the variance in climate change discussion within the high climate change perception group. Notably, social norms emerged as the strongest predictor in this model. After accounting for social norms and self-efficacy, the predictive power of response efficacy weakened considerably, with perceived others' response efficacy not being a significant predictor of climate change discussion.

### 3.3. Factors influencing climate change discussions in groups with low climate change perceptions

Similarly, using multilevel linear regression analysis and a stepwise approach, we examined factors influencing climate change discussions within the group characterized by low climate change perception. In Model 1, the influence of sociodemographic variables on climate change discussions was assessed (Table 4). The results showed that Income 1 had a significant negative association with climate change discussion. Specifically, individuals with a monthly income below 2,000 Yuan were more likely to engage in climate change discussions (β = −0.44, se = 0.64, *p* = 0.001).

**Table 4:** Factors influencing climate change discussions in groups with low climate change perceptions.

	**Model 1**			**Model 2**			**Model 3**		
	**β**	**SE**	***p***	**β**	**SE**	***p***	**β**	**SE**	***p***
Gender	−0.03	0.19	0.47	−0.02	0.15	0.49	−0.04	0.11	0.14
Age	0.00	0.01	0.96	−0.04	0.01	0.29	−0.04	0.01	0.16
Place of residence	−0.02	0.30	0.60	−0.01	0.24	0.75	−0.01	0.18	0.85
Income 1	−0.44	0.64	0.00	−0.35	0.51	0.00	−0.16	0.38	0.04
Income 2	−0.22	0.63	0.09	−0.18	0.50	0.09	−0.08	0.37	0.31
Income 3	−0.13	0.62	0.39	−0.17	0.49	0.14	−0.13	0.36	0.14
Income 4	−0.16	0.65	0.13	−0.16	0.51	0.05	−0.11	0.38	0.07
Education 1	0.05	0.80	0.28	0.00	0.64	0.99	0.03	0.47	0.31
Education 2	0.12	0.48	0.06	0.04	0.38	0.47	0.01	0.28	0.77
Education 3	0.09	0.35	0.19	0.02	0.28	0.64	0.06	0.20	0.14
Personal response efficacy				0.23	0.08	0.00	0.06	0.06	0.18
Perceived others' response efficacy				0.41	0.07	0.00	0.07	0.06	0.13
Personal efficacy							0.31	0.04	0.00
Social norm							0.48	0.04	0.00
R^2^	0.05			0.39			0.70		
F	4.86			36.68			113.16		

β is standardized; SE represents standard error.

In Model 2, after controlling for sociodemographic variables, we explored the impact of response efficacy on climate change discussions. The findings indicated that both perceived personal (β = 0.23, se = 0.08, *p* < 0.001) and others' response efficacy (β = 0.41, se = 0.07, *p* < 0.001) positively predicted climate change discussions, with others' response efficacy having a larger effect size. In Model 3, building upon Model 2, we investigated the effects of self-efficacy and social norms on climate change discussions. The results revealed that both self-efficacy (β = 0.31, se = 0.04, *p* < 0.001) and social norms (β = 0.48, se = 0.04, *p* < 0.001) had a positive influence on climate change discussions. However, neither perceived personal response efficacy nor perceived others' response efficacy had significant predictive effects (*p* > 0.05).

In summary, the full model explained 70% of the variance in climate change discussion within the low climate change perception group. The key finding suggests that social norms remained the most influential factor, while the predictive role of response efficacy was not significant.

## 4. Discussion

In this study, we conducted a comprehensive survey with a large sample size to investigate the impact of response efficacy and social norms on climate change discussions from a social interaction perspective. Based on the findings supporting our hypotheses, the following conclusions can be drawn.

Firstly, we observed that groups with high climate change perceptions had more frequent climate change discussions compared to groups with low climate change perceptions. This suggests that individuals who possess sufficient scientific knowledge about climate change, such as believing in human-caused climate change, are more willing to engage in climate change discussions with their family and friends. This finding aligns with previous research (Tian et al., 2022), highlighting the pivotal role of climate change perceptions as a driver of climate change discussions.

Secondly, in groups with high climate change perceptions, self-efficacy, response efficacy, and social norms emerged as the primary factors influencing climate change discussions, jointly explaining 69% of the variation. Notably, social norms were found to be the most influential factor. While previous studies have focused on the psychological mechanisms of socially constructed silence (Geiger and Swim, 2016), they have overlooked the influence of social norms on climate change discussions. From a social interaction perspective, we argue that social norms reflect individuals' assessment of others' ability to act, which influences their decision to discuss climate change. Additionally, we found that personal response efficacy, rather than the perceived others' response efficacy, influenced climate change discussions in groups with high climate change perceptions. This indicates that individuals in these groups are primarily concerned with the benefits of the discussion for themselves, such as enhancing their own knowledge and skills, when empowering others to participate in climate change discussions. Although the response efficacy of others did not facilitate discussions, it is noteworthy that the high climate change perceptions group was motivated to discuss climate change irrespective of whether it benefitted others. This is promising because individuals who prioritize climate action encourage collective engagement and are not hindered by learned helplessness (Maier and Seligman, 1976).

Lastly, in groups with low climate change perceptions, self-efficacy and social norms were the main drivers of participation in climate change discussions. Social norms remained the most influential factor, underscoring their significance in climate change discussions. However, the role of response efficacy, whether personal or others', was not significant. This aligns with our expectations that groups with low climate change perceptions are concerned about their own abilities and the abilities of those around them to engage in climate change discussions. Put simply, if they lack confidence in their own and others' capacities for climate change discussions, such discussions will not occur. For the low climate change perception group, limited scientific understanding of climate change and possible misconceptions about the attitudes of others regarding climate change undermine their trust in their own and others' abilities. Consequently, high self-efficacy and strong social norms promote their engagement in climate change discussions. Regarding response efficacy, they do not prioritize whether the discussion benefits themselves or others due to their low expectations of the outcome. This finding is consistent with previous research (Geiger et al., 2017), emphasizing the importance of individuals' perceived ability to act compared to the impact of behavior.

In summary, from a social interaction perspective, individuals' assessment of others (i.e., social norms) emerged as a decisive factor influencing climate change discussions, surpassing the significance of self-assessment found in previous studies. Moreover, individuals' assessment of competence in climate change discussions had a greater impact on these discussions compared to assessments of the potential impact, encompassing both competence assessments (self-efficacy and social norms) and impact assessments (perceived personal response efficacy and perceived others' response efficacy).

Our findings carry several implications for the theoretical literature and intervention efforts related to climate change discussions. First, we propose exploring the psychological mechanisms of climate change discussions from a social interaction perspective, which offers fresh insights into the field. While prior research has examined the relevance of self and the social construction of climate silence (Geiger and Swim, 2016; Geiger et al., 2017), there remains a need for systematic investigations into the influence of individuals' assessments of self and others on climate change discussions from an integrated standpoint. Thus, our study contributes to the theoretical advancement of climate change discussions. Second, we found that social norms play a pivotal role in driving climate change discussions in both high and low climate change perceptions groups, highlighting the importance of considering individuals' assessment of others in social interactions. This finding provides communicators with novel strategies to encourage public participation in climate change discussions. Similar to previous research (Schultz, 1999), communicators can leverage information about social norms to facilitate engagement in these discussions among the public. Lastly, individual assessments of competence, such as self-efficacy and social norms, exerted a greater influence on climate change discussions than assessments of behavior. This evidence supports the notion that educators can cultivate individuals' capacity to participate in climate change discussions, thereby fostering such discussions and overcoming barriers associated with learned helplessness.

However, it is important to acknowledge several limitations of our study. Firstly, although we conducted a large sample survey to assess the impact of response efficacy and social norms on climate change discussions from a social interaction perspective, the results remain correlational and lack causal arguments. We encourage future research to investigate the influence of climate change discourse from a social interaction perspective through laboratory experiments, which can provide more robust evidence and establish causal relationships. Secondly, while our study sheds light on the psychological mechanisms underlying people's engagement in climate change discussions, it is necessary to develop effective intervention strategies that can encourage and facilitate such discussions. Future research should focus on designing interventions based on psychological factors and evaluating their effectiveness and long-term impact in promoting climate change discussions.

In conclusion, our study contributes valuable insights into the role of response efficacy and social norms in climate change discussions from a social interaction perspective. It emphasizes the importance of individuals' assessments of others and their own competence in driving these discussions. Our findings have implications for theoretical advancements in understanding climate change discussions and provide guidance for communicators and educators in promoting public engagement in climate change conversations. However, further research utilizing experimental methods and intervention strategies is needed to deepen our understanding and develop practical approaches to foster climate change discussions.

## Data availability statement

The raw data supporting the conclusions of this article will be made available by the authors, without undue reservation.

## Ethics statement

The studies involving humans were approved by the Institute of Psychology, Chinese Academy of Sciences. The studies were conducted in accordance with the local legislation and institutional requirements. The participants provided their written informed consent to participate in this study.

## Author contributions

JT: Conceptualization, Data curation, Formal analysis, Methodology, Writing—original draft. XZ: Conceptualization, Data curation, Investigation, Visualization, Writing—original draft. YS: Funding acquisition, Resources, Supervision, Validation, Writing—original draft.

## References

[B1] Bandura A. (1977). Self-efficacy: toward a unifying theory of behavioral change. Psychol. Rev. 84, 191–215. 10.1037/0033-295X.84.2.191 847061

[B2] Bandura A. (1982). Self-efficacy mechanism in human agency. Am. Psychol. 37, 122–147. 10.1037/0003-066X.37.2.122

[B3] Bandura A. (1986). Social Foundations of Thought and Action: A Social Cognitive Theory. New York, NY: Prentice–Hall.

[B4] Bandura A. (1997). Self-Efficacy: The Exercise of Control. New York, NY: Freeman.

[B5] Berkhout F. Hertin J. Jordan A. (2002). Socio-economic futures in climate change impact assessment: using scenarios as ‘learning machines'. Global Environ. Change 12, 83–95. 10.1016/S0959-3780(02)00006-7

[B6] Bonan J. Cattaneo C. d'Adda G. Tavoni M. (2020). The interaction of descriptive and injunctive social norms in promoting energy conservation. Nat. Energy 5, 900–909. 10.1038/s41560-020-00719-z

[B7] Boudet H. Giordono L. Zanocco C. Satein H. Whitley H. (2020). Event attribution and partisanship shape local discussion of climate change after extreme weather. Nat. Climate Change 10, 69–76. 10.1038/s41558-019-0641-3

[B8] Bouman T. Verschoor M. Albers C. J. Böhm G. Fisher S. D. Poortinga W.. (2020). When worry about climate change leads to climate action: How values, worry and personal responsibility relate to various climate actions. Global Environ. Change 62, 102061. 10.1016/j.gloenvcha.2020.102061

[B9] Cialdini R. B. Jacobson R. P. (2021). Influences of social norms on climate change-related behaviors. Curr. Opin. Behav. Sci. 42, 1–8. 10.1016/j.cobeha.2021.01.005

[B10] Cialdini R. B. Reno R. R. Kallgren C. A. (1990). A focus theory of normative conduct: Recycling the concept of norms to reduce littering in public places. J. Person. Soc. Psychol. 58, 1015–1026. 10.1037/0022-3514.58.6.1015

[B11] Clayton S. Devine-Wright P. Stern P. C. Whitmarsh L. Carrico A. Steg L.. (2015). Psychological research and global climate change. Nat. Climate Change 5, 640–646. 10.1038/nclimate2622

[B12] Doherty K. L. Webler T. N. (2016). Social norms and efficacy beliefs drive the alarmed segment's public-sphere climate actions. Nat. Climate Change 6, 879–884. 10.1038/nclimate3025

[B13] Geiger N. Swim J. K. (2016). Climate of silence: pluralistic ignorance as a barrier to climate change discussion. J. Environ. Psychol. 47, 79–90. 10.1016/j.jenvp.2016.05.002

[B14] Geiger N. Swim J. K. Fraser J. (2017). Creating a climate for change: Interventions, efficacy and public discussion about climate change. J. Environ. Psychol. 51, 104–116. 10.1016/j.jenvp.2017.03.010

[B15] Goldberg M. H. van der Linden S. Maibach E. Leiserowitz A. (2019). Discussing global warming leads to greater acceptance of climate science. Proc. Nat. Acad. Sci. 116, 14804–14805. 10.1073/pnas.1906589116 31285333 PMC6660749

[B16] Heath Y. Gifford R. (2002). Extending the theory of planned behavior: predicting the use of public transportation. J. Appl. Soc. Psychol. 32, 2154–2189. 10.1111/j.1559-1816.2002.tb02068.x

[B17] Hoegh-Guldberg O. Jacob D. Taylor M. Guillén Bolaños T. Bindi M. Brown S.. (2019). The human imperative of stabilizing global climate change at 1, 5. C. Science 365, eaaw6974. 10.1126/science.aaw6974 31604209

[B18] IPCC. (2021). Climate Change 2021: The Physical Science Basis. Contribution of Working Group I to the Sixth Assessment Report of the Intergovernmental Panel on Climate Change. Cambridge: Cambridge University Press.

[B19] Kashima Y. (2014). Meaning, grounding, and the construction of social reality. Asian J. Soc. Psychol. 17, 81–95. 10.1111/ajsp.12051

[B20] Leiserowitz A. Carman J. Buttermore N. Neyens L. Rosenthal S. Marlon J.. (2022). International Public Opinion on Climate Change, 2022. New Haven, CT: Yale Program on Climate Change Communication and Data for Good at Meta.

[B21] Leiserowitz A. Maibach E. Rosenthal S. Kotcher J. Bergquist P. Ballew M.. (2020). Climate Change in the American Mind: November 2019. Yale University and George Mason University. New Haven, CT: Yale Program on Climate Change Communication. 10.31234/osf.io/z3wtx

[B22] Leiserowitz A. Maibach E. Roser-Renouf C. Feinberg G. Rosenthal S. (2015). Climate Change in the American Mind: March, 2015. New Haven, CT: Yale University and George Mason University.

[B23] Leiserowitz A. Maibach E. W. Rosenthal S. Kotcher J. Bergquist P. Ballew M.. (2019). Climate Change in the American Mind: April 2019. New Haven, CT: Yale University and George Mason University.

[B24] Leung K. Morris M. W. (2015). Values, schemas, and norms in the culture-behavior nexus: a situated dynamics framework. J. Int. Bus. Stu. 46, 1028–1050. 10.1057/jibs.2014.66

[B25] Liu X. Chen S. Guo X. Fu H. (2022). Can social norms promote recycled water use on campus? The evidence from event-related potentials. Front. Psychol. 13, 818292. 10.3389/fpsyg.2022.818292 35185735 PMC8856723

[B26] Lynas M. Houlton B. Z. Perry S. (2021). Greater than 99% consensus on human caused climate change in the peer-reviewed scientific literature. Environ. Res. Lett. 16, 114005. 10.1088/1748-9326/ac2966

[B27] Maier S. F. Seligman M. E. (1976). Learned helplessness: theory and evidence. J. Exp. Psychol. General 105, 3–46. 10.1037/0096-3445.105.1.3

[B28] Morris M. W. Hong Y. Y. Chiu C. Y. Liu Z. (2015). Normology: Integrating insights about social norms to understand cultural dynamics. Org. Behav. Hum. Dec. Proc. 129, 1–13. 10.1016/j.obhdp.2015.03.001

[B29] Noelle-Neumann E. (1991). The theory of public opinion: the concept of the spiral of silence. Annal. Int. Commun. Assoc. 14, 256–287. 10.1080/23808985.1991.1167879035452372

[B30] Noelle-Neumann E. (1993). The Spiral of Silence: Public Opinion-Our Social Skin. Chicago: University of Chicago Press.

[B31] Nolan J. M. Schultz P. W. Cialdini R. B. Goldstein N. J. Griskevicius V. (2008). Normative social influence is underdetected. Pers. Soc. Psychol. Bullet. 34, 913–923. 10.1177/0146167208316691 18550863

[B32] Patz J. A. Campbell-Lendrum D. Holloway T. Foley J. A. (2005). Impact of regional climate change on human health. Nature 438, 310–317. 10.1038/nature04188 16292302

[B33] Plutzer E. McCaffrey M. Hannah A. L. Rosenau J. Berbeco M. Reid A. H.. (2016). Climate confusion among US teachers. Science 351, 664–665. 10.1126/science.aab3907 26912845

[B34] Schneider C. R. van der Linden S. (2023). Social norms as a powerful lever for motivating pro-climate actions. One Earth 6, 346–351. 10.1016/j.oneear.2023.03.014

[B35] Schultz P. W. (1999). Changing behavior with normative feedback interventions: a field experiment on curbside recycling. Basic Appl. Soc. Psychol. 21, 25–36. 10.1207/s15324834basp2101_3

[B36] Sechrist G. B. Swim J. K. Stangor C. (2004). When do the stigmatized make attributions to discrimination occurring to the self and others? The roles of self-presentation and need for control. J. Pers. Soc. Psychol. 87, 111–122. 10.1037/0022-3514.87.1.111 15250796

[B37] Stangor C. Swim J. K. Sechrist G. B. Decoster J. Van Allen K. L. Ottenbreit A.. (2003). Ask, answer, and announce: three stages in perceiving and responding to discrimination. Eur. Rev. Soc. Psychol. 14, 277–311. 10.1080/10463280340000090

[B38] Swim J. K. Fraser J. (2013). Fostering hope in climate change educators. J. Museum Educ. 38, 286–297. 10.1080/10598650.2013.1151078137151292

[B39] Swim J. K. Fraser J. Geiger N. (2014). Teaching the choir to sing: use of social science information to promote public discourse on climate change. J. Land Use Environ. Law 30, 91–117. Available online at: https://www.jstor.org/stable/43741160

[B40] Swim J. K. Geiger N. Fraser J. Pletcher N. (2017). Climate change education at nature-based museums. Curator Museum J. 60, 101–119. 10.1111/cura.1218735199922

[B41] Swim J. K. Geiger N. Sweetland J. Fraser J. (2018). Social Construction of Scientifically Grounded Climate Change Discussions. Psychology and Climate Change. New York, NY: Academic Press, 65–93.

[B42] Tian J. Sun M. Gong Y. Chen X. Sun Y. (2022). Chinese residents' attitudes toward consumption-side climate policy: the role of climate change perception and environmental topic involvement. Res. Conserv. Recycl. 182, 106294. 10.1016/j.resconrec.2022.106294

[B43] United Nations Environment Programme. (2020). Emissions Gap Report 2020. Nairobi, 65-93.

[B44] Van der Linden S. (2017). Determinants and Measurement of Climate Change Risk Perception, Worry, and Concern. The Oxford Encyclopedia of Climate Change Communication. Oxford: Oxford University Press.

[B45] Van Stekelenburg A. Schaap G. Veling H. Buijzen M. (2021). Boosting understanding and identification of scientific consensus can help to correct false beliefs. Psychol. Sci. 32, 1549–1565. 10.1177/09567976211007788 34534026

